# Response to “An Inexpensive Biomechanical Model to Help Teach and Learn Newer Mandible Reduction Techniques”

**Published:** 2023-06-01

**Authors:** Vincent W.M. Lum, Juliana Poh

**Affiliations:** * Changi General Hospital, Department of Accident & Emergency, Singapore, Singapore; † Singapore General Hospital, Department of Emergency Medicine, Bukit Merah, Singapore

Dear Editors,

We thank Tummings et al. for their interest in our article and congratulate them on the use of their innovative mandible reduction trainer. We agree that video demonstrations and hands-on practice are superior to text and static pictures for learning mandibular reduction techniques. Slight variations in techniques may indeed occur with different clinicians as their experience with using these techniques grows.

